# Supplementary material for: Are carbon emissions trading and green financial instruments synergistic? -Comprehensive quantitative research based on content analysis
Source: PLoS One. 2024 Mar 7;19(3):e0298601. doi: 10.1371/journal.pone.0298601 (PMC10919601; doi:10.1371/journal.pone.0298601)
Supplement: S1 Data — (ZIP) [file pone.0298601.s001.zip › Data/地级市Z_F工作报告.zip!/╜¡╬≈/─╧▓2╩╨/└┤╫╘╣1⁄2╓┌║┼í╛┬φ┐╦╩2╛▌═°í┐_2016.docx]

各位代表：

　　现在，我代表南昌市人民政府，向大会作政府工作报告，请予审议，并请各位市政协委员和列席会议的同志提出意见。

　　一、2015年及“十二五”经济社会发展主要情况回顾

　　刚刚过去的2015年，全市上下在省委、省政府和市委的坚强领导下，全面贯彻中央决策部署和省委十六字方针，紧紧围绕市委二十字总体任务，主动适应和把握经济新常态，积极主动稳增长、促改革、调结构、惠民生，全市经济社会保持平稳较快发展势头。

　　过去一年，是经济爬坡过坎、发展提质增效的一年。

　　一是各项主要指标全面完成。预计2015年地区生产总值增长9.6%左右，财政总收入增长13.9%，地方一般公共预算收入增长13.7%，500万元以上固定资产投资增长17%，规模以上工业增加值增长9.3%左右，社会消费品零售总额增长12.5%，利用外资实际到位资金增长16.6%，实际利用内资增长11.7%，海关出口总额增长3.1%，城镇居民人均可支配收入增长9.5%，农村居民人均可支配收入增长10.5%。

　　二是发展质量效益稳步提升。财税收入结构优化，全市税收收入占财政收入比重（市口径）达到91.3%。工业生产效益提升，规模以上工业全员劳动生产率达到33.7万元/人，工业经济综合效益指数超过300%。非公经济活力提升，全市民间投资同比增长26.6%。能源资源消耗下降，万元GDP能耗和电耗分别下降2.1%和2%，万元规模以上工业增加值能耗下降5.9%，用水量下降5.1%。

　　过去一年，是改革持续深入、创新成效显著的一年。

　　一是重点改革进展顺利。企业事中事后监管加快推进，“一处违法、处处受限”的格局基本形成。“三单一网”建设基本完成，达到了“联得上、打得开、有内容”的建设要求。PPP模式融资改革试点加速推进。园区管理体制改革和运行机制改革取得新突破，高新区、经开区、小蓝经开区和临空经济区干部人事制度改革基本完成。不动产登记制度改革基本完成，已经实现全市不动产登记机构、簿册、依据、信息平台“四个统一”。扩权强镇改革试点正式启动，农村产权交易所建设已经完成，在全省率先启动完成统一城乡居民医疗保险制度改革。

　　二是创新驱动成效显著。国家小微企业创业创新基地示范城市申报成功，开局良好，创业创新平台加快搭建，创建省级小微企业创业园14个、创客空间62家，认定第一批“洪城众创”29家。产学研协同创新能力不断增强，硅衬底LED技术荣获国家技术发明一等奖；组建了汽车、半导体照明等12家省级产业技术创新战略联盟，有色金属短流程节能冶金产业技术战略联盟上升为国家级；江西本草天工、江西江中制药“创新药物与高效节能降耗制药设备实验室”升格为国家重点实验室；江西国家数字出版基地正式落户；全年共引进各类高层次人才542名。

　　三是开放合作步伐加快。积极实施“引进来、走出去”战略，组织参加了中博会、赣台会、赣港经贸合作、民企入赣大会等大型招商活动，全年新签约重大项目438个，签约投资额3230亿元；全年新批境外投资企业25家，中方协议投资额6.75亿美元，对外承包工程完成营业额4.25亿美元；对外交流与人员往来共计90万人次。南昌综合保税区申报进展顺利，出口加工区、保税物流园区、昌北国际机场航空口岸等平台更加完善。“昌九一体”、“昌抚一体”合作协调实现常态化，昌抚两市“1+8”合作协议成功签署。《合肥纲要》顺利实施，长江中游城市群省会城市合作再进一步。2015年闽浙赣皖福州经济协作区第十七次市长联席会成功举办。

　　过去一年，是产业加快升级、结构持续优化的一年。

　　一是产业层级快速提升。装备制造、新能源、电子信息等产业快速壮大。C919大飞机中后机身顺利交付，使南昌在世界大飞机制造领域占有了一席之地；中国北车江西轨道交通产业基地填补了我省轨道交通装备产业发展空白，实现了“南昌地铁”南昌制造、南昌维修；百路佳新能源客车已成为海外市场的有力竞争者，江铃新能源汽车取得重大突破；欧菲光二期、中航通信（海派科技）、鸿利光电、酷派（依偎）、凯迅光电等项目密集开工投产，欧菲光触摸屏出货量世界第一，加上对世界500强飞利浦LED芯片和汽车照明事业部80.1%股权的并购，使我市的电子信息产业站到了世界前沿。高新技术产业增加值和战略性新兴产业比重不断提升。全年新增高新技术企业68家，高新技术产业占全市工业增加值的比重达到22%；9个省重点产业集群中，新兴产业集群占比达61.5%，高于全省45.4个百分点。

　　二是产业结构持续优化。服务业占比显著提升，对全市经济增长贡献率超过50%，达到51.8%。金融、电子商务、文化旅游等现代服务业发展迅速。全省金融商务区新增入驻各类金融机构和服务企业41家，全市金融机构本外币存款余额、贷款余额分别增长12.1%、16.3%，区域性金融中心地位日益增强；全市电子商务相关交易额首次突破1000亿元，预计达到1080亿元；新增省级文化产业基地2个，滕王阁景区正式进入5A级旅游景区创建程序，梅岭国家旅游度假区创建工作稳步推进，南昌汉代海昏侯国考古遗址公园建设有序推进，打造全国旅游目的地的步伐明显加快。农业转型升级步伐加快。粮食生产实现“十二连丰”，优质蔬菜基地加快建设，溪霞国家农业综合开发示范园区建设成效明显，休闲农业、都市现代农业加快发展。

　　过去一年，是城乡统筹发展、品质持续提升的一年。

　　一是城市规划更加完善。《南昌大都市区规划》编制初步完成，全市总体规划修编工作启动，历史文化名城保护、传统村落保护、旧城改造等专项规划编制有序开展，新一轮轨道建设规划加快推进，城市综合停车场、综合管廊等服务型重大基础设施建设规划方案编制初步完成。

　　二是城市交通更加便捷。地铁1号线开通运营，昌吉赣城际铁路、地铁2号线及南延线建设进展顺利，地铁3号线正式开工。九龙湖片区内基础路网基本实现通车。象湖隧道、金山大道主线、九洲高架西段竣工通车；沿江南北大道、志敏大道、昌东大道南延等一批城市骨干路网项目启动建设；望城公交枢纽站、瑶北互通立交工程等重大基础设施投入使用；七里岗互通立交、龙头岗综合码头一期主体工程均已完工。105、320国道四个一级公路项目开工建设。

　　三是城乡面貌持续改观。棚户区和旧城改造步伐加快，承担了全省城市棚户区改造任务32505户，东湖区滨江片区、西湖区十字街北街、青云谱区京山老街、青山湖区工人新村等20个项目进展顺利。扎实开展市容环境十二大专项整治和“百项惠民便民利民”工程，完成了34个农贸市场提升改造工程，加快推进了一批老旧社区、街头小游园、社区便利店等与百姓密切相关的生活设施建设或升级改造。18个特色小镇、26个“五位一体”示范村、46个中心村建设加快推进。“清洁家园、清洁水源、清洁田园”工程建设、村民建房规划建设管理专项整治活动、农村“脏乱差”集中整治“百日行动”全面实施。

　　过去一年，是民生不断改善、社会和谐稳定的一年。

　　一是民生状况持续改善。全市新增城镇就业8.32万人，城镇就业率达到95%；新增小额贷款发放11亿元，市本级新增工商登记3.5万户。全市城镇基本养老、医疗、失业、工伤、生育五大保险参保人数分别达到162.8万人、254万人、60.88万人、92.1万人、48.1万人；城乡低保和农村五保集中供养标准继续提高，临时救助制度更加健全；公租房建设任务全面完成。第二期学前教育三年行动计划顺利实施，义务教育“零择校”成效继续巩固，公办高中“零择校”如期实现。基本公共卫生服务经费人均标准继续提高，基层医疗卫生服务网点加快完善，社区卫生服务机构国有化、固定化建设工程顺利实施。养老服务体系进一步完善，新建居家养老服务设施160个。市群艺馆建成开放，完成了20个街道综合文化活动中心标准化建设。完成了16.3万农村人口安全饮水建设工程。

　　二是社会保持和谐稳定。高度重视信访工作，深化平安南昌建设，围绕“两提升”目标，“打、防、整、带、访、宣”六措并举，全市发案数持续下降，破案率继续上升。认真抓好安全生产、消防安全、食品药品安全、应急管理工作，全市没有发生大规模群体性事件、重大治安案件、重特大火灾和安全生产事故。

　　各位代表，2015年主要任务的完成，标志着我市“十二五”规划胜利收官。通过全市上下的共同努力，“十二五”时期，全市经济社会保持持续较快发展，经济实力、城乡发展和人民生活水平发生了可喜变化，为未来五年发展积蓄了巨大能量，积累了宝贵经验。

　　回顾“十二五”，看到的是可喜变化。

　　一是经济实力明显增强。概括来讲就是“三突破、三跨越、三翻番”。“三突破”，就是预计地区生产总值突破4000亿，年均增长11%；人均生产总值突破10000美元，年均增长12%；高新技术产业总产值突破1000亿，年均增长10.9%。“三跨越”，就是财政总收入连续跨越400亿、500亿、600亿、700亿四个台阶，达到727.2亿元，年均增长17.7%；地方一般公共预算收入连续跨越200亿、300亿台阶，达到389.2亿元，年均增长21.6%；农林牧渔业总产值跨上300亿元台阶，年均增长4%。“三翻番”，就是500万元以上固定资产投资实现翻番，达到4000亿元，年均增长20.3%；规模以上工业增加值实现翻番，达到1450亿元，年均增长17.5%；社会消费品零售总额实现翻番，达到1608亿元，年均增长16%。

　　二是城市空间快速拓展。存量空间持续优化，实施了旧城改造三年强攻计划，大力推进棚户区改造工作，完成了两轮“拆违拆临、建绿透绿”行动。累计完成旧城（棚户区）改造面积约110万平方米，拆除违建、临建面积38.3万平方米，还路于民9.99万平方米，还绿于民5.36万平方米。增量空间大幅提升，实施了新城建设三年强攻计划，九龙湖30平方公里核心起步区全面建设，10.35平方公里的朝阳新城基本成型；新建撤县建区顺利完成，长堎镇全面融入中心城区；全市建成区面积比2010年增加77平方公里。临空经济区规划建设快速推进，16.8平方公里的直管区“四横两纵”骨干路网正在加快建设，3平方公里的核心起步区已经建成。空间联系更加紧密，轨道交通加快建设，南昌正式进入地铁时代；“十横十纵”快速路网建成通车42.78公里，正在建设108.22公里；朝阳大桥、象湖隧道等一批过江通道先后建成通车，“一江两岸、南北双城”的发展格局已然形成。

　　三是人民生活显著改善。居民收入大幅提高。城镇居民人均可支配收入达到31855元，比2010年增加13555元，年均增长11.7%；农村居民人均可支配收入13718元，比2010年增加6526元，年均增长13.8%。社会事业快速发展。各类学校发展到2080所，覆盖149.62万名学生，比2010年增加2.28万人，新增幼儿园90所，适龄儿童入学率达到100%。医疗卫生机构974个，比2010年增加176个；全面推进县级公立医院改革，基本药物制度在基层医疗机构实现全覆盖。建成了一批公共图书馆、群艺（文化）馆、博物（纪念）馆、农家书屋、基层文化站和社区文化活动室；3个广播剧、歌剧作品荣获中宣部“五个一工程奖”。成功举办第七届全国城市运动会，建成各类体育场地7706个，人均体育场地面积达2.43平方米。民生投入持续增加。五年累计投入民生工程资金866.3亿元，养老、失业、医疗、工伤和生育保险覆盖率由2010年的95%提高到98%；城乡低保水平提高，城区、县城、农村标准从2010年的300元、260元、120元分别提高到480元、450元、280元。扶贫开发成效显著。全市农村贫困人口由16万人减少到6万人，贫困发生率从6.4%下降到2.5%。生产生活条件明显改善。人口电视覆盖率达到99%，农村道路硬化率达到83.6%。

　　回顾“十二五”，积蓄的是巨大能量。

　　五年来，我们全面推进深化改革，提升开放合作水平，积蓄了巨大的发展动能。始终坚持先行先试，行政审批制度改革、户籍制度改革、投融资体制改革、土地储备制度改革、农村土地承包经营权流转交易市场建设试点工作、失地农民养老保险等重点领域改革走在全国省会城市前列。始终坚持内联外融，省内一小时经济圈初具雏形，南昌大都市区规划建设拉开序幕；与长、珠、闽发达地区和“一带一路”国家战略对接不断加强，引进了汇丰银行控股公司、欧菲光、航天科工、中节能等世界或国内500强企业。

　　五年来，我们全力培育百亿企业、千亿产业和千亿园区，促进产业高位谋势，积蓄了巨大的发展势能。百亿企业由2010年的2家增加到8家；食品产业率先达到千亿规模，汽车及零部件产业接近千亿；高新、经开、小蓝三个国家级开发区主营业务收入全部超过千亿。特别是临空经济区，在短短的一年多时间，不仅路网和标准厂房建设快速推进，一批具有重大引领作用的高端、前端、深端产业项目也快速入驻，其巨大的发展前景已经初露端倪。同时，随着以全省金融商务区为重点载体的现代金融业，以万达文化旅游城、绿地国际博览中心、恒茂梦时代广场、王府井城市综合体和多个电商互联网创业创新项目为主要抓手的现代商贸旅游业的加快发展，现代服务业集聚集群发展势头强劲。粮食等主要农产品连年增收，市级以上农业产业化龙头企业达451家，农业发展稳步走向中高端。

　　五年来，我们稳打稳扎统筹城乡发展，持续优化发展环境，积蓄了巨大的发展潜能。致力于改善农村面貌，共计建设了21个特色镇、72个“五位一体”综合示范村和3581个新农村建设示范点，完成了涉及25条主要干道沿线“五化”综合整治，深入推进了农村生活垃圾处理和农村“清洁家园”工程建设，秀美乡村建设水平快速提升。致力于优化生态环境，持续推进“森林城乡、花园南昌”和“鄱湖明珠·中国水都”建设。开展了“蓝天、清流、净土”行动和象湖、抚河、乌沙河、玉带河等河流湖泊水系整治，完成了艾溪湖森林湿地公园、瑶湖郊野森林公园、大象湖湿地公园、赣江两岸风光带建设。全市集中式饮用水源地水质达标率稳定在100%，主要河流水质达标率达90%以上；空气质量持续优良，其中2015年优良率达到86.3%，位居中部省会城市第一。我市成功入选首批“全国水生态文明城市”建设试点，成功创建了“国家森林城市”。致力于优化社会环境，持续推进法治南昌、平安南昌建设，不断加强和完善城市管理，着力加强和创新社会治理，全社会文明程度明显提升，成功创建“全国文明城市”。

　　与此同时，国防建设、人民防空、拥军优属、国家安全、民族宗教、对台事务、外事侨务等工作持续加强；审计统计、质监安监、参事文史、社科研究、档案史志、机要保密、地名水文、科普气象、老龄慈善、工会、青少年、妇女儿童、残疾人、防灾减灾、爱国卫生、红十字会、机关事务、消费者权益保护等工作取得喜人成绩，为“十二五”经济社会发展起到了巨大的促进和保障作用。

　　回顾“十二五”，积累的是宝贵经验。

　　五年的实践经验表明，做好政府未来五年的工作：

　　要有全新的理念。新常态要有新理念。过去五年，正是因为全市上下主动认识和把握新常态，才使得我们能够应对复杂多变的形势，顶住日益加大的压力，保持了经济社会稳中有进、稳中向好的发展势头。未来五年，我们必须牢牢把握“认识新常态、适应新常态、引领新常态”这个大逻辑，牢固树立“创新、协调、绿色、开放、共享”的发展理念，为全市经济社会发展奠定坚实的思想基础。

　　要有宏大的格局。大龙头要有大格局。要持续深入地“跳出江西看南昌、立足全国看南昌、放眼世界看南昌”，敢于同大的比、与强的争。五年来，我们始终坚持“四个聚焦”，强力推进“大投入、大建设、大发展”，已经让全省、全国看到了南昌站前列、立潮头的新气派，也逐渐显露出了一系列厚积薄发的大气魄。未来五年，我们更要放大格局，以长江中游城市群核心城市、“一带一路”重要节点城市来审视、谋划、推动南昌发展，使南昌真正成为“引领全省发展的排头兵、拉动全省发展的火车头”，真正成为在全国具有重要影响的现代化大都市。

　　要有过人的担当。大时代要有大担当。未来五年，我们国家正处在加快实现第一个百年目标的伟大时代，南昌也正处在打造核心增长极、共筑“四强梦”的关键时期，任务艰巨、使命光荣。我们必须要有一往无前的担当精神，在矛盾、困难和挑战面前不犹豫、不动摇、不退缩，真正做到面对矛盾敢于迎难而上，面对危机敢于挺身而出，面对失误敢于承担责任，面对歪风邪气敢于坚决斗争，努力营造担当可贵、担当光荣的良好氛围。

　　要有恒久的定力。大战略要有大定力。即将提交审议的“十三五”规划纲要草案制定了未来五年的“六大战略”。要切实增强“功成不必在我、功成必定有我”的大局意识，牢固树立持之以恒、久久为功的战略定力，使战略细化为一项一项具体的工作举措，转化为一茬一茬现实的发展成效。

　　要有务实的作风。南昌的地位，说到底是干出来的。通过党的群众路线教育实践活动和“三严三实”专题教育洗礼，以及持续深入的法治政府、廉洁政府、高效政府建设，广大干部的工作作风是过得硬、靠得住的。未来五年，我们要继续保持和弘扬实在实干实绩的优良作风，不耍花招、不图虚名，狠抓落实、务求实效，为“十三五”规划目标顺利实现提供有力的作风保障。

　　过去五年，我们虽然取得了很大的成就，但仍要看到，南昌经济总量还不够大，经济实力还不够强，属于欠发达省会城市的基本市情没有根本改变；“不进则退、慢进也是退、稍有松懈就会掉队”的竞争格局没有根本改变；经济运行结构性矛盾和深层次问题尚未解决，不少影响发展的体制机制障碍还有待进一步突破；有核心竞争力的产业和企业还不够多，新的经济增长动力、增长板块还不够强；城市承载功能和管理水平有待加强，节约资源和保护环境任务还很重；保障和改善民生压力不小，政府效能仍需进一步提高。对此，我们将采取切实措施，认真加以解决。

　　各位代表，五年的发展成就来之不易，五年的发展经验弥足珍贵。这些成绩和经验的取得，归功于省委、省政府和市委的正确领导，归功于市人大、市政协的监督指导，归功于历届班子打下的坚实基础，归功于各民主党派、工商联、无党派人士、各人民团体和驻昌单位、人民解放军、武警官兵以及离退休老同志的大力支持，归功于全市广大干部群众的团结一心、奋力拼搏。在此，我谨代表市人民政府，向全市人民，向所有关心、支持南昌发展的同志们、朋友们，表示衷心的感谢并致以崇高的敬意！

　　二、关于“十三五”规划纲要(草案)的主要内容和2016年工作安排

　　“十三五”时期是我市打造核心增长极、实现“四个强起来”的关键阶段，也是我市全面建成小康社会的决胜阶段。按照《中共南昌市委关于制定全市国民经济和社会发展第十三个五年规划的建议》要求，《纲要(草案)》提出了“十三五”时期全市经济社会发展的指导思想：高举中国特色社会主义伟大旗帜，以马列主义、毛泽东思想、邓小平理论、“三个代表”重要思想和科学发展观为指导，深入贯彻习近平总书记系列重要讲话精神，全面落实省委“发展升级、小康提速、绿色崛起、实干兴赣”十六字方针，牢牢把握“打造核心增长极基本成型”主线，奋力开创“抢占制高点、聚焦增长极、提升首位度、共筑‘四强’梦”新局面，确保在全省率先全面建成小康社会，努力把南昌建设成为工业文明、城市文明、生态文明融合发展的现代化城市。

　　按照上述指导思想，综合考虑宏观发展形势和我市基本市情，《纲要(草案)》提出了全市“十三五”经济社会发展的主要目标：到2020年，实现“两提前一成型”，建设“六个南昌”。即提前实现2020年较2010年人均生产总值和城乡居民收入翻番，提前并在全省率先全面建成小康社会；打造核心增长极基本成型；建设实力南昌、创新南昌、开放南昌、智慧南昌、美丽南昌和文明南昌。力争实现人均生产总值20000美元，地区生产总值占全省比重达到1/4左右；工业主营业务收入10000亿元；战略性新兴产业增加值、服务业增加值和500万元以上固定资产投资规模翻番；农村居民可支配收入增加10000元，建设整洁舒适、生态宜居城镇。

　　为完成上述目标，《纲要(草案)》制定了六大主要战略、七大总体布局和五项重大举措。六大主要战略分别是，赶超升级战略、创新驱动战略、开放协调战略、生态优化战略、品牌引领战略和共享发展战略；七大总体布局分别是，主体功能布局、城镇体系布局、优势产业布局、城乡人口布局、区域合作布局、公共服务布局和重大时序布局；五项重大举措分别是，厚植高新高端的产业优势、建设揽山临湖的山水都市、打造开放创新的发展高地、提升共享发展的生活品质、增强省会治理的能力水平。

　　各位代表，2016年是全面建成小康社会决胜阶段的开局之年，也是推进结构性改革的攻坚之年。

　　做好今年的工作，必须坚持党的领导，全面贯彻党的十八大、十八届三中、四中、五中全会、中央经济工作会议，以及省委十三届十二次全会和市委十届十二次全会精神，牢固树立和贯彻落实创新、协调、绿色、开放、共享发展理念，紧紧围绕市委二十字总体任务，主动适应和把握经济新常态，以提质、增效、升级为中心，以改革、创新、开放为动力，着力稳定经济增长，全力主攻产业发展，加快打造城市品牌，不断强化辐射带动，持续提升民生福祉，努力实现“十三五”时期经济社会发展良好开局。

　　今年经济社会发展的主要预期目标是：地区生产总值增长9.5%；财政总收入增长10%；地方一般公共预算收入增长10%；全社会固定资产投资增长16%；规模以上工业增加值增长9.5%左右；社会消费品零售总额增长12%；利用外资实际到位资金增长10%；实际利用内资增长8%；出口总额增长2%左右；城镇居民人均可支配收入增长9.5%；农村居民人均可支配收入增长10%；万元生产总值能耗下降2%；居民消费价格总水平涨幅控制在3%左右。

　　今年我们将主要抓好以下工作：

　　1.做好充分的思想准备和政策储备，突出优供给扩需求，着力稳定经济增长。

　　2016年，南昌发展的外部环境错综复杂，经济下行压力还很大。我们要有充分的思想准备和政策储备，既打好持久战，又打好攻坚战，着力稳定经济增长。

　　一是持续优化供给。优产出，加快供给结构升级。通过设立专项资金、发展服务业吸纳就业等手段，转型升级一批不符合国家能耗、环保、质量、安全等标准和长期亏损的过剩供给；通过技术改造、市场创新等手段，优化生产流程，提升产品质量，转型升级一批纺织服装、机电等传统产业供给；通过鼓励创新创业、放开准入限制，强化产品创新、业态创新，加快战略性新兴产业发展步伐，创造一批符合时代要求、适应消费需求的全新供给。降成本，积极破解企业难题。大力开展“优化企业发展环境”和“企业大走访”活动，为企业解难事、办实事。进一步落实和完善《关于稳增长促发展的若干政策措施》，全面梳理政府层面为企业减轻负担的政策措施，降低企业制度性交易成本、企业税费负担、社会保险费以及财务融资成本，协调降低电力等成本。继续推广“财园信贷通”、财政惠农信贷通等融资模式，完善融资担保体系，扶持中小企业转贷公司发展，着力解决企业融资难融资贵问题。补短板，全面保障要素供给。人力资源方面，深入实施各类引才育才工程，着力保障高层次、高技能人才需求；进一步消除人口城乡流动门槛，提高户籍人口城镇化率；全面落实二孩政策，持续稳定劳动力要素供给。土地资源方面，加大闲置土地资源处置力度，发挥近几年储备土地作用，全面盘活土地存量；加大土地整理力度，加快农村土地经营权确权流转，大幅提升土地增量。资金要素方面，通过股权多元化改革、完善治理结构、转型经营方式、创新融资方式，持续做大做强投融资平台；通过系统性整合与“募、投、管、退”全过程市场化运作，做大做强产业发展基金，不断提升产业基金对产业发展的扶持力度和引导水平；加快发展股权交易市场、债券市场，探索设立生产要素交易平台，加快推进企业上市、上市公司再融资和“新三板”挂牌工作。

　　二是全力扩大需求。积极扩大有效投入。大力实施总投资3400亿元的基础设施、产业升级、公共服务等领域的164个重大重点项目。着力推动PPP模式改革，放开民间资本投资领域，引导更多的民间资金参与到基础设施、公共服务领域中来。加快释放消费需求。积极发展住房租赁市场，实现公租房货币化，有效释放住房刚性需求和改善性需求。积极推动皮卡等轻型货车下乡，落实小排量汽车、新能源汽车税收优惠政策，扩大汽车消费。加快智慧城市建设，推动网络提速降费，促进信息消费。切实落实带薪休假制度，鼓励实行错峰休假、弹性作息，推动旅游消费。大力发展养老家政健康消费，扩大教育文化体育消费。进一步营造良好消费环境，让人民群众能消费、敢消费。全力稳定出口增长。积极扶持进出口企业转型升级，引导企业通过新型贸易方式培育技术、品牌、质量、服务为核心的竞争新优势。大力支持企业参加“一带一路”沿线国家博览会，引导企业开拓国际市场，将南昌商品推向世界。

　　2.抢抓难得的时间窗口和战略机遇，突出优平台强集群，着力构建以战略性新兴产业为主导的现代产业体系。

　　目前南昌正处于传统产业升级的拐点期、战略性新兴产业茁壮成长的窗口期。我们必须以时不我待的紧迫感，抢抓难得的战略机遇，高处谋势、新处落子，把传统产业转型升级、战略性新兴产业发展壮大的好苗头，转化为全市产业向中高端快速迈进的好势头。

　　一是以高新高端为目标、转型升级为动力，全力打造一批现代制造业集群。充分发挥硅衬底LED技术优势，组建市光电产业研究院和国家级检测中心，加快建设南昌LED产业创新示范园区，进一步完善电子信息产业布局，全力打造“南昌光谷”。以经开区新能源汽车项目和小蓝经开区汽车项目为依托，加快出台促进新能源汽车产业基地建设的政策措施，着力打造南昌汽车城。以C919大飞机、中直公司合作项目、大洋通航总部及运营基地项目为依托，加快中航工业洪都商用飞机、南昌通用飞机、航空科技开发及其他相关产业发展，着力打造南昌航空城。以江中药谷、济民可信、汇仁制药为龙头，尽快出台支持生物医药产业发展的政策措施，加大全市生物医药企业集聚、产业整合力度，着力打造南昌生物医药城。

　　二是以创业创新为依托、文化底蕴为支撑，全力打造一批现代服务业集群。继续发挥江西慧谷·红谷创意产业园、江西樟树林文创产业园、699文化创意园、小蓝创业创新基地等园区平台作用，全力打造“南昌慧谷”。利用“互联网＋”,积极发展众创、众包、众扶、众筹等新模式,加快文化创意、游戏动漫、电子商务、服务外包等新兴服务业态集聚发展。深入推进全省金融商务区和金融产业服务园区建设，力争到2016年底引进金融机构（企业）总数达到200家左右，建设区域性金融中心。以大型城市综合体和特色商业街为依托，重点建设八一广场中山路、红谷滩新区两大市级商业中心，规划建设9个区级商业中心。着力推进向塘物流产业集群等8个省级物流产业集群建设，加快申报2016年国家城市物流标准化试点项目，着力构建现代高效物流服务体系。充分发挥南昌文化底蕴深厚的优势，组建南昌文化旅游产业基金，鼓励和引导社会资本投资文化产业，以万达文化旅游城项目正式运营、南昌汉代海昏侯国考古遗址公园建设、滕王阁国家5A级旅游景区和梅岭国家旅游度假区创建等为契机，努力打造一批文化旅游新亮点，进一步唱响绿博会等会展品牌，让文化旅游会展成为南昌新的经济增长点。

　　三是以精品示范为引领、绿色有机为特色，全力打造一批都市现代农业集群。坚持“做精品、强示范”，强力推进“两区一廊”建设，确保规划核心区域范围内基础设施、配套设施及江西省现代农业博览园、赣鄱珍奇植物园等相关重点项目基本建成，努力唱响“百年乡村画、千年田野风”品牌，打造“南昌绿谷”。充分发挥江西绿色有机产品种类多、品牌响的特色优势，做强做优一批农业产业化龙头企业，力争全市产值10亿元以上的龙头企业达到16家；统筹推进粮食加工、农产品物流等七大产业集群建设。

　　为推进产业集群发展，必须抓好公共服务和开发区平台建设。公共服务平台方面，通过建设扶持、认定奖励、采购服务等方式，加快建设研发、检测、设计、融资、展销、检验检疫、物流通关、报税结算等区域性、专业化、开放型产业公共服务平台，为企业提供优质完善的公共服务，为产业集群发展提供更多便利。开发区平台方面，加快高新区产城融合步伐，提升生态品牌和在全国的地位，促进高新区向2000亿元台阶跨越；加快经开区进位赶超步伐，全面实施主营业务收入“三年翻番”计划；促进小蓝走绿色发展、集约发展、创新发展之路，争创传统产业发展转型示范区、新兴产业创新创造集聚区、体制机制改革先行区，全力打造小蓝经开区升级版。加快推进长江经济带国家级产业转型升级示范开发区长堎工业园区申报步伐。

　　3.激发强烈的忧患意识和行动自觉，突出抓改革促创新，着力培育经济增长新动力。

　　新常态下，不改革就不能前进，不创新就不能升级，不开放就不能生存。我们将不断增强“不日新者必日退”的忧患意识和敢为天下先的改革意识，奋力闯出一条发展新路。

　　一是深入推动改革攻坚。深化简政放权，继续推进行政审批制度改革，深化商事制度改革，建立健全企业事中事后监管制度，抓好监管系统的运行和部门的联合执法，让失信企业“一处违法、处处受限”；深入推进“三单一网”建设，推动政府权责清单管理和便民服务取得实实在在的成效。完善体制机制，进一步理顺临空经济区管理体制，着力消除临空经济区发展的体制机制障碍；积极创新重点产业投融资体制机制，大力发展多元结构的产业引导基金，着力推动县区、开发区（新区）参与重点产业投融资体系；继续完善公共资源交易平台和监管体制。消除市场壁垒，深入推进新一轮国资国企改革，促进国有资本更多向中高端产业集中；优化金融生态环境，依法维护金融债权，加强金融监管，规范扶持小贷公司和融资性担保公司发展；进一步消除进入门槛，放宽投资领域，坚定不移促进非公经济的发展。

　　二是提升创新驱动能力。着力打造创新创业平台。健全研发平台，深化与高等院校、科研院所合作，加快建设大学科技园。扎实推动国家小微企业创业创新基地城市示范三年行动计划，力争今年新增一批科技企业孵化器，大力打造一批“洪城众创”空间。发展高新产业，力争今年新增高新技术企业75家，培育科技小巨人企业60家。完善服务体系，鼓励支持众创服务平台建设，加快启动南昌科技创新服务基地建设，高标准建设南昌国家知识产权示范城市。强化创新创业要素支撑。全面整合全市各类科技资金，引导社会资本参与，大力支持企业技术创新和科技成果转化。加快推进科技金融建设步伐，为科技创新提供便捷、快速的信贷、保险、担保支持。营造创新创业氛围。努力营造“人人都能创业、个个都可创新”的浓厚氛围，让每个人都可以把创业创新的冲动转化为行动,把创业创新的构想付诸于实践,进而找到施展自身才华、实现自身价值的用武之地，使南昌成为创业创新之城。

　　三是全面提升开放水平。切实提升理念、视野和格局。贯彻落实开放发展理念，必须要有国际化视野，坚持国内和国际两个市场统筹、利用外资与对外投资并重、引资和引技引智结合，加快促进要素有序流动、资源高效配置、市场深度融合。尤其要创新招商理念，真正把着力点放在优化服务、健全发展环境、维护政府信用和形成高端要素配套上。大力建设口岸、平台和通道。加快建设完善昌北国际机场航空口岸、综合保税区、出口加工区、保税物流中心、加工贸易梯度转移承接基地。积极对接中欧国际班列，力争开通全省首条欧洲航线；以高等级航道和快速铁路建设为重点，进一步打通连接长江水道和出海的通道；探索与“一带一路”国家和地区的通信便利化，努力拓展陆上、海上、空中、数字四大开放通道。着力强化招商、贸易和合作。始终抓牢“一号工程”，大力开展产业招商、专业招商、精准招商、商会招商、协会招商、以商招商。大力拓展国际市场，支持企业在“一带一路”沿线国家和地区建设国际营销网络和售后服务体系；组织引导龙头企业开拓“一带一路”沿线国家和地区工程市场，着力打响“南昌建设”品牌。全面加强合作交流，推进与“一带一路”沿线国家和地区的合作交流，积极发展友好城市关系；加强国内区域合作，深度融入长江中游城市群，着力加强与闽浙赣皖福州经济协作区、泛珠三角省会城市等协作区的经贸合作和高层互动；深入推进昌九、昌抚一体化，推进昌九在更高层次、更宽领域开展合作，与抚州市共同研究编制昌抚合作示范区规划。全面完善体制、机制和政策。积极争取国家“构建开放型经济新体制综合试点城市”，主动对接、借鉴复制上海、天津、广东、福建自贸区试点经验；不断完善南昌市重点产业投资引导资金运行管理机制，进一步强化招商引资决策协调、调度落实和督查考核机制。

　　4.坚持系统统筹思维和以人为本理念，突出强功能促协调，着力打造城市品牌。

　　城市发展的核心是人，我们将坚决贯彻中央城市工作会议精神，遵循城市发展规律，着力推进城乡一体化发展。

　　一是坚持系统统筹，完善城市规划。坚持城市用地与人口相匹配、城市规模与资源环境承载能力相适应的原则，推进新一轮城市总体规划和城市综合交通规划修编。以主体功能区规划为基础，统筹各类空间性规划，推进“多规合一”。完成《南昌市城市公共空间专项规划》、《扬子洲地区概念规划》、《南昌市综合管廊规划》、《南昌市燃气专项规划》等编制工作。

　　二是坚持以人为本，健全城市功能。加快城市建设步伐，提升城市管理水平，进一步健全城市功能。交通方面，加快轨道2、3号线建设，继续推进“十横十纵”干线路网、城市跨江跨湖通道、城市出口通道建设，大力实施道路桥梁维修改造；实施“主城区道路交通畅通工程”，持续开展“五车”、“六乱”整治行动；启动实施“停车场三年攻坚计划”，力争三年内我市主城区“停车难”问题有所缓解。管网方面，加快推进城市综合管廊建设，着力建设“海绵城市”；继续推进“杆线下地”工程，尽力消除城市“空中线网”。公共服务方面，全面完成九龙湖新城外国语学校初高中、腾龙学校、南昌十中经开校区、前湖小学及部分幼儿园建设；加快推进省妇幼保健院、省二附院、省文化中心项目建设，开展古豫章十景的挖掘以及历史文化街区的恢复工作。生活配套方面，继续大力推进惠民便民利民工程，强化社区环境整治，推进小游园、便利店建设和农贸市场提升改造；新建一批城区公厕、垃圾中转站，进一步加强背街小巷及重点部位补充照明。

　　三是坚持协调发展，促进城乡一体。继续推进18个特色小镇打造；结合开展“六好农户、六好村庄”创建、“千万树木进千村”等工作，加大新农村村点整治和村民建房管理力度，大力推广农村新户型，建设一批示范村，推动我市新农村建设迈上新台阶。

　　5.树立绿色理念和底线思维，突出优生态创示范，着力提升资源环境承载能力。

　　生态是南昌的核心竞争力之一，必须以对历史负责、对人民负责、对后代负责的高度责任感，全面做好建设、保护、治理各项工作。

　　一是倡导绿色理念。始终坚持“生态立市、绿色发展”的指导思想，守住生态底线，大力倡导绿色低碳行为，推行绿色生产生活方式，积极引导绿色消费、节约消费、适度消费。

　　二是坚守生态红线。加快制定《南昌市生态保护红线区划》，探索建立完善资源环境承载能力监测预警机制、生态环境损害补偿修复制度，在我市重点生态区域探索推进生态补偿试点。

　　三是强化建设治理。围绕“森林城乡、花园南昌”、“鄱湖明珠·中国水都”以及全国生态文明先行示范区建设，持续推进生态建设，强化保护治理。加强生态建设，继续推进赣东大堤风光带景观工程、玉带河园林景观改造提升工程、八大山人梅湖景区改造提升工程、赣抚尾闾综合整治工程和九龙湖综合整治工程等重大项目；支持湾里区、新建区开展生态文明先行示范区建设。强化保护治理，加大环保执法力度，让破坏生态环境的“任性”行为付出沉重代价；深入实施“大气、水、土”三大行动，加强大气污染跨区域联防联控，力争空气质量优良天数超过300天；加强城镇生活污水、工业园区污水处理设施及配套管网建设和管理，严格控制排污总量，全力推进“河湖长制”试点工作，力争城市集中式饮用水源地水质达标率达到100%；强化土壤污染源头综合治理，严格控制农业面源污染。

　　6.恪守坚定的责任意识和为民情怀，突出惠民生强治理，着力提升群众幸福指数。

　　百姓的关注点，就是政府工作的着力点，不断增强群众的获得感、认同感、安全感是我们义不容辞的责任，也是我们坚持不懈的追求。

　　一是切实增强群众获得感。千方百计促进收入增长，力争新增城镇就业6.87万人，新增农村劳动力转移就业3.3万人。全面落实企业职工工资正常增长机制，增加居民工资性收入；积极出台调整工薪政策，提高城乡居民收入水平。精准把脉推进扶贫攻坚，全力抓好80个扶贫开发工作重点村的整村推进，大力实施“十到户七进村”精准扶贫工程，深入推进“百名书记扶百村、千个单位挂千点、万名干部联万户”帮扶行动，全面开展“光伏扶贫”工作。持续深入加强社会保障，稳妥推进城乡居民医疗保险制度改革，进一步提高低保、抚恤标准和养老金发放水平。统筹城乡医疗救助工作，全面启动养老服务业综合改革。多措并举加强住房保障，按时完成公租房基本建成任务，及时启动完成2015、2016年度公共租赁住房分配工作，尽快制定已购经济适用住房上市和回购操作流程。

　　二是切实增强群众认同感。深化公立医院改革，完善农村医疗卫生服务网络和城市社区医疗卫生服务体系，健全分级诊疗制度，着力破解看病难。继续实施第二期学前三年行动计划，实施大学区改革和中小学校长、老师轮岗制度，推进义务教育优质化发展，着力破解上学难。完成10个街道综合文化活动中心建设，举办“书香南昌”、“首届南昌马拉松大赛”等活动。

　　三是切实增强群众安全感。深化矛盾纠纷排查化解，坚持依法信访治访；加强社会治安防控体系建设，完善“天网”体系，加大社会治安排查整治力度，打造一批“平安建设示范村（社区）”，强化反恐维稳工作；深入开展食品药品安全专项整治和质量提升行动；全力加强社会诚信建设；继续加强安全生产管理和应急管理，全力维护社会和谐稳定。

　　各位代表，发展的最终目的是为了人民，我们将更加努力加大民生投入、优化公共服务、强化社会治理，让全体市民过得更加顺心、更加幸福。

　　政府的能力和作风是做好工作的重要保障。我们将进一步加强政府自身建设。一是强力推进依法行政。把公众参与、专家论证、风险评估、合法性审查、集体讨论决定作为重大决策的必经程序，严格按照法定权限和程序开展工作，全面规范行政行为。二是全面推进政务公开。加大政府信息公开力度，全面整合、优化各类非紧急类及公共服务热线，加快建设统一权威、便民高效的12345政府服务热线平台。三是深入推进作风建设。深入贯彻“一准则两条例”，巩固“三严三实”专题教育成果，严格落实中央八项规定精神。开展“为官不为，为政不廉”常态化治理，深化“红包”、“小金库”和领导干部违规插手干预工程项目等问题的专项治理，加大惩治腐败和纪律约束力度，坚决遏制腐败滋生。

　　各位代表，回首过去五年，我们豪情满怀；展望未来发展，我们信心百倍。让我们紧密团结在以习近平同志为总书记的党中央周围，在省委、省政府和市委的正确领导下，坚定信心、团结拼搏、创新进取、扎实苦干，努力实现“十三五”良好开局，为打造核心增长极、共筑“四强梦”作出新的更大贡献！
